# “Molecular pigeon” network of lncRNA and miRNA: decoding metabolic reprogramming in patients with lung cancer

**DOI:** 10.3389/fonc.2025.1578927

**Published:** 2025-07-17

**Authors:** Mingxiao Li, Ling Dai, Simin Chen, Minjie Deng, Lihuai Wang, Yinhui Sun

**Affiliations:** ^1^ Medical School, Hunan University of Chinese Medicine, Changsha, China; ^2^ Oncology Medical Center, The First Hospital of Hunan University of Chinese Medicine, Changsha, China

**Keywords:** lncRNA, miRNA, metabolic reprogramming, NSCLC, lung cancer

## Abstract

In recent years, with the advancement of RNA analysis techniques, such as single-cell RNA sequencing, noncoding RNAs have demonstrated substantial potential in regulating gene expression, encoding peptides and proteins, constructing the cellular microenvironment, and modulating cell function. They can serve as potential therapeutic targets and diagnostic markers for various diseases, offering novel avenues for diagnosis and treatment. Among them, long noncoding RNAs (lncRNAs) represent a principal component. Through the competing endogenous RNA mechanism, lncRNAs sequester microRNAs (miRNAs), interact with metabolic enzymes or transcription factors, regulate gene expression, and participate in the metabolic communication network within the tumor microenvironment. This process significantly promotes the growth, proliferation, and metastasis of lung cancer cells by reprogramming core metabolic pathways—including glucose utilization, lipid homeostasis, and amino acid flux. This article reviews the key roles of lncRNAs and miRNAs in the metabolic reprogramming of patients with lung cancer, elucidates the complex lncRNA–miRNA network involved, and provides mechanistic insights into metabolic vulnerabilities and translational opportunities for targeted interventions in the diagnosis and treatment of lung cancer.

## 1 Introduction

Lung cancer remains the most prevalent malignant neoplasm worldwide and presents substantial public health challenges, as evidenced by its disproportionate mortality burden. According to statistics, lung cancer accounts for approximately 18.4% of total cancer-related deaths. Despite advancements in diagnostic and therapeutic technologies, the 5-year survival rate for patients with primary lung cancer remains below 16%, posing a persistent global public health concern (1, 2). As research delves deeper into the biological characteristics of tumors, the hallmark framework proposed by Hanahan and Weinberg has been continually refined, with metabolic reprogramming emerging as a key feature enabling tumor cells to adapt to microenvironmental stress and sustain malignant phenotypes (3). This metabolic remodeling extends beyond the classical Warburg effect (enhanced aerobic glycolysis) to encompass multidimensional regulatory networks, including the dynamic balance of oxidative phosphorylation (OXPHOS), lipid metabolic reprogramming, and amino acid metabolic dysregulation (4–13).

As a vital member of the noncoding RNA family, research on long noncoding RNAs (lncRNAs) has undergone a paradigm shift from being considered “transcriptional noise” to being recognized as “functional regulators.” Since their discovery in 1976, the biological functions of lncRNAs have gradually become clearer (14). By the early 21st century, high-throughput sequencing technologies revealed numerous lncRNA expression profiles (14). Since then, their roles have been systematically analyzed and characterized (14–20), gradually unveiling the complexities of lncRNA biology. LncRNAs are extensively involved in constructing tumor metabolic networks through multiple mechanisms, including the competitive endogenous RNA (ceRNA) mechanism, protein binding, and transcriptional or posttranscriptional regulation of gene expression. For example, LINC01123 enhances glycolytic flux by stabilizing c-Myc transcripts; AC020978 binds to pyruvate kinase M2 (PKM2) to simultaneously drive glycolysis and metastasis; and ADPGK-AS1 modulates M2 macrophage polarization via exosomes, thereby reshaping the tumor microenvironment (TME) (21–25). These findings highlight the pivotal role of lncRNAs in tumor metabolic reprogramming and introduce novel molecular targets and research paradigms for the diagnosis and treatment of patients with lung cancer.

Here, we systematically review the lncRNA–miRNA regulatory networks involved in the metabolic reprogramming of patients with lung cancer, focusing on their molecular mechanisms in glucose, lipid, and amino acid metabolism. In addition, we integrate relevant research from the past decade to explore: (i) how lncRNAs regulate rate-limiting metabolic enzymes through the ceRNA mechanism; (ii) the dynamic influence of lncRNA–protein interactions on metabolic pathways; and (iii) the interplay between metabolic microenvironment remodeling and tumor progression. Furthermore, individualized diagnostic and therapeutic strategies based on lncRNA expression profiles have progressed to phase II clinical trials (NCT04808362) (26), suggesting substantial translational potential. The objective of this review is to provide a theoretical foundation for understanding metabolic heterogeneity in patients with lung cancer and for developing precise, targeted intervention strategies.

## 2 Overview and development of lncRNA

LncRNAs are noncoding transcripts greater than 200 nucleotides in length. In 1976, research on lncRNAs was first documented, and early investigations predominantly characterized their universality and basic features. In the early 21st century, the ENCODE project employed systematic mapping technologies to identify numerous previously unknown lncRNAs and to establish their critical roles in gene regulation (14). Subsequently, various studies have been conducted to deepen our understanding of lncRNAs through data analysis and feature profiling (1–5, 14–19). LncRNAs are not restricted to the nucleus; they are also widely found in the cytoplasm, mitochondria, endoplasmic reticulum, ribosomes (where they affect protein translation), the cell periphery (associated with cell adhesion and connectivity), and exosomes (which influence cancer cell growth, metastasis, or innate immune activation) (27). LncRNAs exhibit high tissue and cell specificity, and their functions are modulated by subcellular localization, abundance, and interacting molecules (27–29). The growing body of knowledge surrounding lncRNAs has deepened the scope of related research, leading to increasing recognition of their roles in cell proliferation, migration, DNA repair, chromatin organization, and the pathogenesis of numerous diseases (29–38), thereby addressing critical gaps in prior research.

Since 2020, lncRNA-focused research has expanded rapidly, with more than 50% of studies concentrating on cancer. Other investigated areas include autophagy, subcellular localization and function, fibrosis, aging, immunity to COVID-19 infection, cardiovascular disease, diabetes, mental illness, and ischemia–reperfusion injury (27, 39–45). In recent years, many researchers have integrated multi-omics networks and machine learning to enhance the efficiency and accuracy of predicting lncRNA–disease associations using big data (46–49). The rapid evolution of artificial intelligence technology and its integration into medical research have revitalized modern scientific inquiry.

## 3 Significance of metabolic reprogramming in tumor progression

In the human body, cells primarily generate energy through several metabolic pathways: OXPHOS, glycolysis, lipid oxidation, and amino acid metabolism. In tumor cells, these metabolic processes undergo phenotypic transformation. In recent years, dynamic shifts between glycolytic reprogramming (Warburg effect) and OXPHOS activation in cancer cells have spurred a surge of research. Warburg first identified abnormal energy metabolism in tumor cells in 1930, referring to the phenomenon whereby tumor cells preferentially undergo glycolysis even in the presence of adequate oxygen. This shift satisfies their rapid energy and biosynthetic demands by enhancing glucose uptake and lactic acid production (8). Although subsequent studies have confirmed this observation (50, 51), several questions remain. These include the limited effectiveness of glycolysis-targeted therapies (52), high OXPHOS levels in some tumors (7, 53), and the presence of metabolic heterogeneity in tumors (54). Additionally, the reverse Warburg effect describes how stromal cells within the TME support tumor growth by secreting metabolites and promoting angiogenesis (52).

Over decades of study, the metabolic characteristics of tumor cells have gradually become clearer. Hanahan and Weinberg’s seminal 2000 (3) framework outlining six hallmarks of cancer was later expanded in 2011 to include two enabling characteristics (genomic instability and tumor-promoting inflammation) and two new hallmarks: energy metabolism reprogramming and immune evasion. Metabolic reprogramming refers to the capacity of tumor cells to reconfigure their energy networks—particularly glycolysis and lipid metabolism—to adapt to microenvironmental stress and sustain malignant phenotypes (8).

OXPHOS is the cellular process whereby reduced equivalents (NADH and FADH_2_) generated from the oxidation of organic substrates (including glucose and fatty acids) are converted into ATP through the synergistic action of the electron transport chain and ATP synthase (55). OXPHOS can support rapid tumor proliferation by efficiently producing ATP. Additionally, intermediates from the tricarboxylic acid (TCA) cycle, such as α-ketoglutarate and oxaloacetate, provide carbon backbones for synthesizing lipids, nucleic acids, and amino acids—thereby fulfilling the biosynthetic demands of proliferating tumor cells (56). While the Warburg effect dominates many solid tumors, OXPHOS plays an essential role in leukemia, drug-resistant tumor cells, and cancer stem cells (57). Metabolic plasticity allows tumors to adapt to microenvironmental stressors, such as hypoxia or nutrient deprivation. For example, under hypoxic conditions, HIF-1α promotes glycolysis by upregulating GLUT1 and lactate dehydrogenase A (LDHA), while suppressing OXPHOS (58). Conversely, cancer stem cells often rely on OXPHOS for self-renewal, driven by PGC-1α and mitochondrial biogenesis (59). Which metabolic process—OXPHOS or the Warburg effect—plays a more crucial role in tumor progression (including invasion, proliferation, and drug resistance) remains unknown. Interestingly, pancreatic ductal adenocarcinoma cells exhibit a mixed metabolic phenotype, simultaneously employing glycolysis and OXPHOS via glutamine-driven TCA cycle activity (60). In addition, metabolism in lung tumor cells does not shift from OXPHOS to glycolysis but instead involves enhancement of both pathways (54). In light of this, Liu et al. demonstrated that OXPHOS is upregulated during the dissemination of breast cancer and remains active during colonization, while aerobic glycolysis persists—indicating that the metabolic landscape of tumor cells exhibits complex spatial and temporal heterogeneity that warrants further investigation.

The metabolic pattern transition of tumor cells is dynamically regulated by various molecular networks, involving many processes, such as carcinogenic signals, epigenetic modifications, and microenvironment interactions. For instance, BRAF inhibitors abnormally activate OXPHOS in melanoma through the MITF–PGC1α axis, thereby promoting drug resistance (61). Dual inhibition strategies targeting these adaptive mechanisms, such as combining the glycolysis inhibitor 2-DG with the OXPHOS inhibitor metformin, have shown synergistic therapeutic potential (62). In addition, Hu et al. found that Sirt5 knockout enhances glutamine and glutathione metabolism through acetylation-mediated GOT1 activation, thereby promoting the progression of KRAS mutant pancreatic cancer, and metabolic competition within the TME also shapes metabolic phenotypes. For instance, tumor cells can overtake glucose by overexpressing the glucose transporter GLUT1, resulting in glucose deprivation in the microenvironment and forcing adjacent immune cells (such as T cells) to become dysfunctional owing to insufficient energy, thus avoiding immune surveillance (63). In addition, lactic acid, as a major byproduct of glycolysis, is secreted extracellularly through monocarboxylate transporters (MCT1/MCT4), taken up by cancer-associated fibroblasts (CAFs), and used for OXPHOS to form a ‘lactic acid shuttle’ that sustains tumor growth (64). The competitive consumption of glutamine also significantly affects the metabolic phenotype. Tumor cells deplete glutamine in the microenvironment by upregulating the glutamine enzyme (GLS1), inhibiting macrophage M1 polarization, and promoting the tumor-promoting M2 phenotype (65). Exosome-mediated metabolite delivery, such as succinic acid, can activate HIF-1α signaling and induce chemotherapy resistance (62).

In summary, metabolic reprogramming endows malignant cells with unique abilities, including cell growth and proliferation at multiple levels (9–11), metastasis (12, 13), drug resistance (4, 5), immune regulation (6), and TME remodeling (7). For example, KRAS and LKB1 mutant non-small cell lung cancer (NSCLC) cells synthesize pyrimidines by upregulating CPS1 to provide raw materials for proliferation (9); lipid metabolism reprogramming in hepatocellular carcinoma alters cell membrane fluidity and mediates migration activity (12); when chemotherapy-sensitive lung adenocarcinoma cells are transformed into drug-tolerant persister cells, this transformation is closely related to PINK1-mediated mitophagy, enhanced OXPHOS, and cellular redox imbalance (5).

## 4 LncRNA, miRNA, and lung tumor metabolic reprogramming

Lung cancer is the most common malignant tumor worldwide, accounting for 18.4% of total cancer mortality. Despite advancements in diagnostic and therapeutic technologies, the 5-year survival rate of primary lung cancer is less than 16%, representing a considerable global health challenge (1, 2). Lung cancer is mainly divided into small cell lung cancer (SCLC) and NSCLC. NSCLC accounts for 85% of all cases, including lung adenocarcinoma and squamous cell lung cancer. Although the incidence of SCLC is lower than that of NSCLC, it increases rapidly and carries a high risk of metastasis (5). Aberrant expression of certain lncRNAs in lung cancer cells, such as HOTAIR, MALAT1, and HOTTIP (66–70), is closely related to TNM stage, lymph node metastasis, and survival rate. Conversely, other lncRNAs, including MEG3, LINC01537, and MIR17HG, are downregulated in tumor tissues, suggesting that they may inhibit tumor development (71–73). Current research on lncRNAs in cancer has primarily focused on their roles in reprogramming cancer cell metabolism (21), regulating the tumor immune microenvironment (22), promoting cancer cell invasion and metastasis (23, 24), and interacting with proteins (25). This article discusses the regulation of lncRNAs in the metabolic reprogramming of lung tumor cells.

The ceRNA hypothesis was originally proposed by Leonardo et al. (74), which posits that the interaction between RNAs is based on microRNA binding sites (microRNA response elements) and constructs a ceRNA network with potential effects on diseases. Emerging tools, such as CRISPR-based screening and multi-omics integration, are decoding ceRNA interactions in specific contexts, providing new directions for biomarkers (such as serum lncRNA HOTAIR) and therapeutic targets.

**Figure 1:**
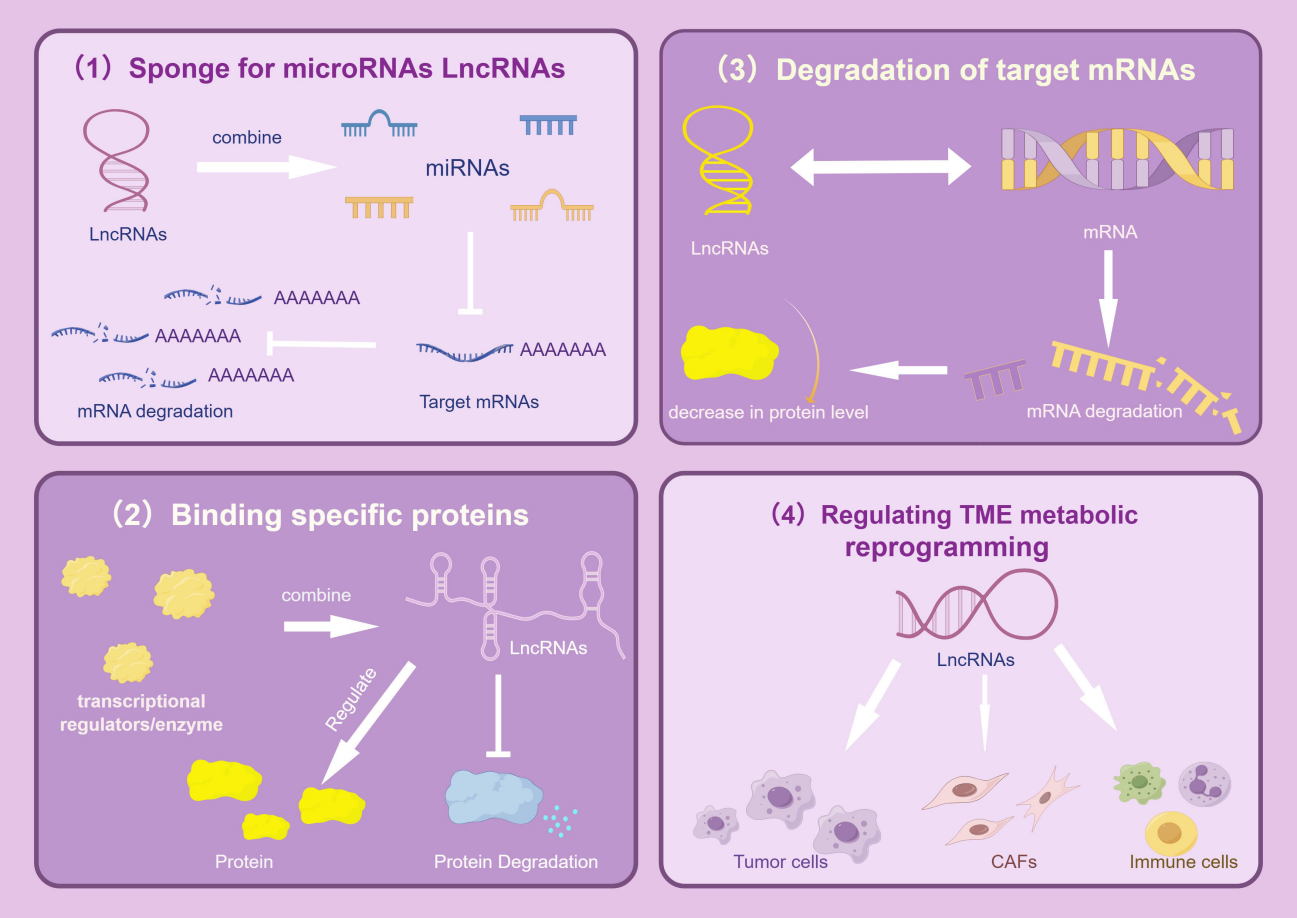
Regulatory mechanism of LncRNAs. The picture mainly describes the four main gene expression regulation mechanisms of LncRNAs in tumors, which are: As a ceRNAs “molecular sponge”, it adsorbs miRNAs, affects the enrichment level of specific proteins, promotes the degradation of target mRNAs, and regulates TME metabolic reprogramming, thereby mediating complex and accurate regulatory mechanisms and significantly promoting the growth, proliferation and metastasis of lung cancer cells. The image is created with Figdraw.

In most cases, cells use glucose and other carbohydrates to produce energy. Therefore, studies on lncRNA-mediated metabolic reprogramming of lung tumors have predominantly focused on modulating cell glucose metabolism. By summarizing existing research findings, we conclude that the mechanism of lncRNA regulating metabolic reprogramming of tumor cells mainly includes (1): acting as a ceRNA ‘sponge’ to adsorb miRNAs, thereby protecting mRNA translation from miRNA interference (2); combining with key enzymes to improve their stability and adjust the content of metabolic enzymes; (3) interacting with transcription factors to indirectly regulate gene expression; (4) participating in metabolic remodeling of the TME; and (5) regulating gene expression at the posttranscriptional level (as shown in **Figure 1**).

### 4.1 Glucose metabolism

**Figure 2:**
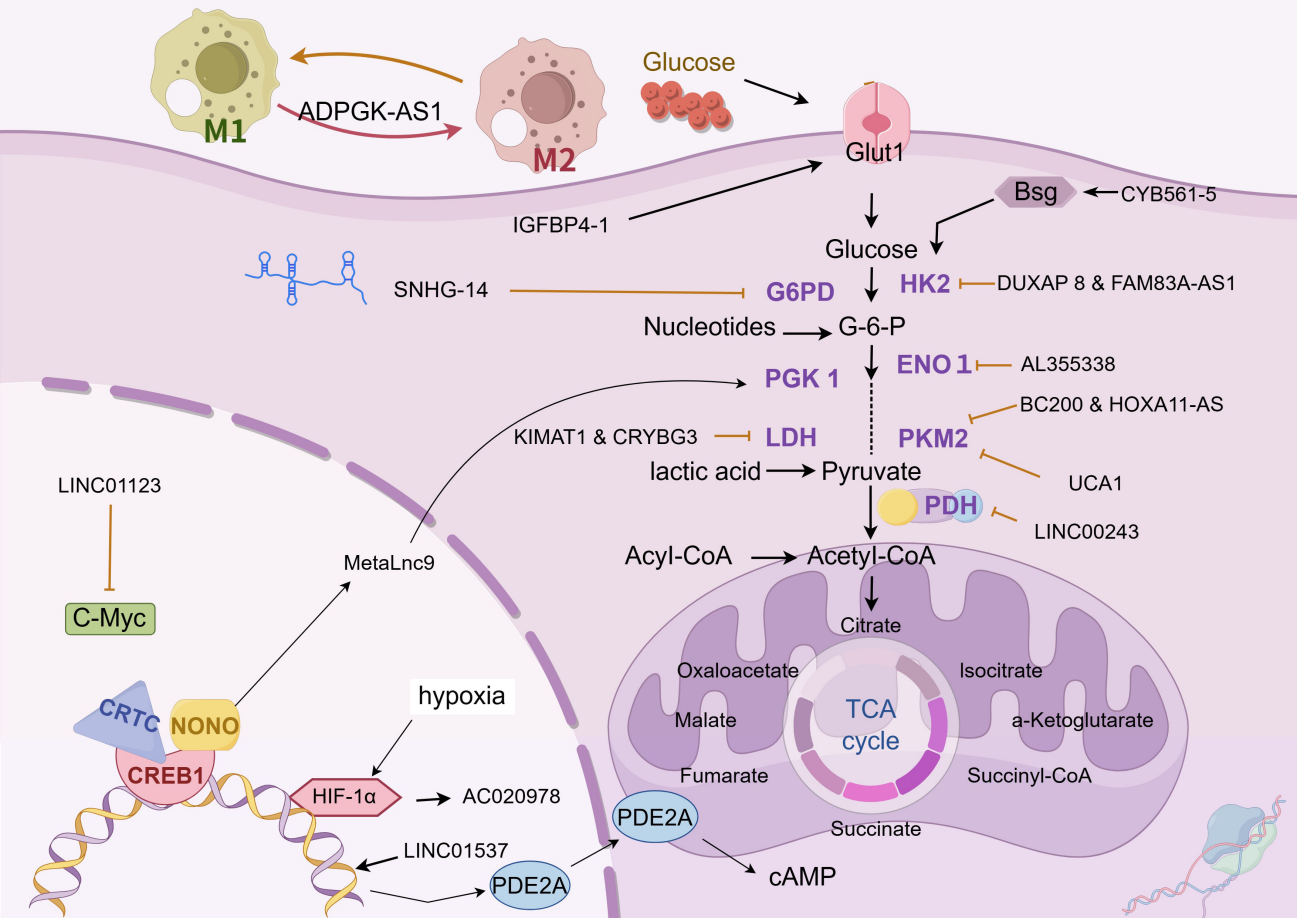
Glucose metabolism. The chart illustrates the regulation mechanism of different LncRNAs on glucose metabolism in lung cancer cells. These lncRNAs influence the abundance of glucose metabolic enzymes, glucose uptake, and the expression of related transcription factors through various pathways, in order to adapt to their rapid growth, metastasis and immune escape. The following is a supplement to the specific mechanisms in the figures: CYB561-5: Interacts with Bsg protein and stimulates the expression of HK2 and PFK1; DUXAP8 & FAM83A-AS1: Upregulate HIF-1α and glycolysis-related enzymes, and target miR-203-3p to boost HK2 expression; AL355338: Binds with ENO1 to enhance cellular glycolysis and drive malignant evolution of NSCLC via EGFR - AKT.; BC200 & HOXA11-AS: Function as a molecular sponge for miR-148b-3p to promote PKM2 expression; LINC00243: Sequesters miR-507 to enhance PDK4 expression; lnc-IGFBP4-1: Mainly localizes in the nucleus, regulates the transcription of glycolysis - related genes like GLUT1, PKM2, and HK2, and increases the expression of glycolysis - associated enzymes; SNHG14: Acts as a molecular sponge for miR-206, upregulates G6PD expression, and helps tumor cells combat oxidative stress, participate in antioxidant synthesis, and generate free radicals; KIMAT1 & LncCRYBG3: Activate mTOR to promote MYC transcription, and bind with LDH; LINC01123 competes with miR-199a-5p to relieve the suppression of c-Myc mRNA, and also participates in the progression of lung adenocarcinoma via the ZEB1/LINC01123/miR-499b-5p/NOTCH1 axis; MetaLnc9: Forms a NONO/CRTC/CREB1 complex in the nucleus to interfere with transcription, and binds with PGK1 in the cytoplasm to prevent its degradation and activate the Akt/mTOR pathway; AC020978: Under the regulation of HIF-1α binding site in the upstream region, it is inversely regulated by hypoxic conditions and HIF-1α . It binds to and stabilizes PKM2, modulates the interaction between PKM2 and HIF-1α to enhance the transcriptional activity of HIF-1α , and also binds to MDH2 to activate the PI3K-AKT pathway; LINC01537, mainly located in the cytoplasm, stabilizes PDE2A mRNA, enhancing its expression, and thus inhibits mitochondrial respiration and the Warburg effect. When its expression is down - regulated in lung cancer cell mitochondria, PDE2A expression decreases correspondingly, regulating mitochondrial biological activity and the Warburg effect. The image is created with Figdraw.

Glucose metabolism is the key transformation in tumor metabolic reprogramming. Tumor cells need to ingest and metabolize carbon sources, such as glucose, to meet the needs of rapid growth, metastasis, and immune escape. This process involves adjustments in the type, distribution, and abundance of cellular metabolic enzymes, indicating the presence of complex and precise regulatory mechanisms at the genetic level (as shown in **Figure 2** and **Table 1**).

**Table 1:** Glucose metabolism.

LncRNA	Targets	Downstream component	Deregulation	Functions	References
LINC01123	miR-199 a-5 p	c-Myc/HK/LDHA	up	strengthen glycolysis	(75)
LINC01123	miR-449b-5p	NOTCH1	up	strengthen glycolysis	(76)
DUXAP 8	miR-409-3p	HK2/LDHA	up	strengthen glycolysis	(77)
HOTAIR	miR-498	ABCE1	up	numerous	(66)
BCYRN1 (BC200)	miR-149	PKM2	up	strengthen glycolysis and metastaisis	(78)
LINC00243	miR-507	PDK4	up	strengthen glycolysis	(79)
HOXA11-AS	miR-148b-3p	PKM2	up	strengthen glycolysis	(79)
LINC00365	miR-429	KCTD12	up	strengthen glycolysis	(80)
AP000695.2	miR-335-3p	TEAD1	up	strengthen glycolysis and metastaisis	(81)
AC016727.1	miR-98-5p	BACH1/HIF-1 α	up	strengthen glycolysis and metastaisis	(82)
LINC00511	miR-625-5p	PKM2	up	promote invasion and migration	(83)
MALAT1	miR-613	COMMD8	up	strengthen glycolysis	(84)
HOXA11-AS	miR-3619-5p	SALL4	up	strengthen glycolysis	(85)
SNHG14	miR-206	G6PD	up	promote the viability	(86)
HOTTIP	miR-615-3p	HMGB3	up	promote hypoxia-induced glycolysis	(68)
AC020978	PKM2	HIF-1α	up	strengthen glycolysis	(87)
CYB561-5	Bsg	HK2/PFK1	up	strengthen glycolysis	(88)
AL355338	ENO1	EGFR-AKT	up	strengthen glycolysis	(89)
KIMAT1	AMPKα	LDHB	up	suppress AMPKα phosphorylation status	(90)
MetaLnc9	mTOR	PGK1	up	promote migration and invasion	(91)
FAM83A-AS1	miR-203-3p	HK2	up	strengthen glycolysis	(92, 93)
lnc-IGFBP	IGFBP4	GLUT1/PKM2/G6PDH/PDK1	up	strengthen glycolysis	(94)
UCA1	mTOR	unknown	up	strengthen glycolysis	(95)
ABHD11-AS1	EZH2;KLF4	unknown	up	promote the Warburg effect	(96)
LINC01537	PDE2A mRNA	unknown	down	attenuated Warburg effect and mitochondrial respiration	(97)
ADPGK-AS1	numerous	numerous	up	Macrophage phenotype transformation	(98)
Mir100hg	miR-15a-5p/miR-31-5p	unknown	up	strengthen glycolysis	(99)
OIP5-AS1	miR-142-5p/miR-200c-3p	PD-L1/GPC4	up	immune escape;strengthen glycolysis	(100, 101)

#### 4.1.1 LncRNA acts as a molecular sponge to regulate miRNA

LncRNAs, such as LINC01123, DUXAP8, BCYRN1, LINC00243, HOXA11-AS, MALAT1, SNHG14, and FAM83A-AS1, are significantly upregulated in lung cancer tissues and are closely associated with tumor stage, lymph node metastasis risk, and poor prognosis (66, 69, 75, 77, 78, 85, 102, 103). These lncRNAs regulate glucose metabolism in lung cancer cells through diverse mechanisms and promote tumor progression. LINC01123 induces abnormal proliferation of lung adenocarcinoma cells, increases the expression and activity of key glycolytic enzymes, and enhances glucose uptake, lactate production, oxygen consumption, and ATP generation (75). Mechanistically, LINC01123 removes the inhibition of c-Myc mRNA by miR-199a-5p through competitive binding, creating a positive feedback loop and participating in lung adenocarcinoma progression via the ZEB1/LINC01123/miR-499b-5p/NOTCH1 axis (76). DUXAP8 upregulates the expression of key glycolytic enzymes by binding to miR-409-3p (77); BCYRN1 is mainly expressed in actively proliferating cells and enhances glycolysis and lactate secretion through the c-Myc/BCYRN1/miR-149/PKM2 axis (78); LINC00243 captures miR-507 and promotes the expression of PDK4 (79); HOXA11-AS, highly expressed in lung adenocarcinoma cells, acts as a molecular sponge for miR-148b-3p, upregulates PKM2 expression, and regulates glucose metabolism reprogramming (79); MALAT1 targets miR-613 in NSCLC, downregulates COMMD8 expression, and enhances cell proliferation and glycolysis (84); as a molecular sponge for miR-206, SNHG14 upregulates G6PD expression, helping tumor cells resist oxidation and participate in reductive synthesis (86); FAM83A-AS1 promotes tumor cell metastasis and invasion in hypoxic environments by upregulating HIF-1α and glycolysis-related enzymes (92). Additionally, lncRNAs can target miR-203-3p to increase HK2 expression, thereby enhancing glycolysis and exacerbating the progression of lung adenocarcinoma (93).

#### 4.1.2 LncRNA binding protein

LncRNAs, such as AC020978, CYB561-5, LncCRYBG3, AL355338, KIMAT1, and MetaLnc9, are highly expressed in lung cancer tissues and are closely associated with tumor stage and poor prognosis (87–89, 91, 104, 105). These lncRNAs alter the metabolic pattern of lung cancer cells and promote tumor progression through multilevel regulation. AC020978 is reversibly regulated by hypoxic conditions and HIF-1α because of a HIF-1α binding site in its upstream region. It binds to and stabilizes PKM2, prolongs its functional activity, regulates its interaction with HIF-1α, enhances HIF-1α transcriptional activity, affects the expression of glucose metabolism-related proteins, and promotes cell proliferation and glycolysis (87). AC020978 also binds to malate dehydrogenase 2 (a mitochondrial enzyme involved in the TCA cycle and the malate–aspartate shuttle, MDH2), activates the PI3K–AKT pathway, and promotes lung cancer cell metastasis (106). CYB561-5 interacts with basigin to stimulate the expression of hexokinase 2 (the first rate-limiting enzyme in glycolysis, HK2) and phosphofructokinase-1 (the second rate-limiting enzyme in glycolysis, PFK1), thereby affecting glycolysis (88). LncCRYBG3 binds specifically to LDHA, a key glycolytic enzyme, and promotes glycolysis (107). AL355338 enhances glycolysis by binding to enolase 1( ENO1 ) and promotes the malignant evolution of NSCLC via the EGFR–AKT pathway (89). KIMAT1 promotes MYC transcription by activating mTOR; together with LDHB, it enhances glycolysis and the TCA cycle, thereby promoting tumor growth (90). MetaLnc9 forms a NONO/CRTC/CREB1 complex in the nucleus, interfering with transcription; in the cytoplasm, it binds to phosphoglycerate kinase 1 and prevents its degradation, thereby enhancing glycolysis and activating the Akt/mTOR pathway to promote cell metastasis (91).

#### 4.1.3 LncRNA regulates gene expression at transcriptional or posttranscriptional levels

Some lncRNAs regulate gene expression via transcriptional or posttranscriptional mechanisms. The different binding targets determine the distinct modes of action between the two: transcriptional mechanism refers to the regulation of target gene activity at the transcriptional initiation stage by directly binding to chromatin modification complexes or transcription factors, whereas posttranscriptional mechanism refers to the regulation of RNA stability, splicing, or translation efficiency through binding to mRNA or miRNA. The expression of lnc-IGFBP4-1 and ABHD11-AS1 is upregulated in lung cancer tissues, whereas LINC01537 is downregulated; these expression patterns are associated with tumor stage and poor prognosis (92, 94, 95, 97, 108). These lncRNAs regulate the metabolic reprogramming of lung cancer cells through various mechanisms and contribute to tumor progression. Among them, ABHD11-AS1 inhibits KLF4 transcription by recruiting the histone methyltransferase EZH2 to its promoter region, contributing to epigenetic regulation (108). Lnc-IGFBP4-1, which is predominantly localized in the nucleus, promotes cell growth, proliferation, and glycolytic metabolism by regulating the transcription of glycolytic genes, such as *GLUT1*, *PKM2*, and *HK2* (94). This lncRNA increases the expression of glycolysis-related enzymes, including GLUT1, PKM2, G6PDH, HK2, PDK1, and LDHA, at the transcriptional level, thereby increasing intracellular ATP production (94). However, the specific regulatory mechanisms remain poorly understood. The increased expression of ABHD11-AS1 in NSCLC tissues is mainly attributed to methylation modification by METTL3 (108). ABHD11-AS1 is enriched in the nucleus and functions as a transcriptional cofactor to regulate gene expression (108). LncRNAs can also recruit EZH2 to the promoter region of KLF4 and inhibit its expression (108). In addition, ABHD11-AS1 can interact with SART3 to alter its subcellular localization, thereby indirectly regulating RNA splicing and promoting malignant transformation and tumor stem cell growth (96). LINC01537, primarily located in the cytoplasm, stabilizes phosphodiesterase 2A mRNA, thereby increasing its expression and inhibiting both mitochondrial respiration and the Warburg effect (97). Downregulation of LINC01537 in the mitochondria of lung cancer cells leads to a corresponding decrease in phosphodiesterase 2A expression, impaired mitochondrial function, and alterations in the Warburg effect, thereby inhibiting cell death (97).

#### 4.1.4 Metabolic communication network involved in the regulation of the TME

The TME refers to the local tissue environment established by tumor cells to facilitate their growth, proliferation, and migration. Its cellular components include non-immune cells (such as tumor cells, CAFs, and endothelial cells) and immune cells (such as tumor-associated macrophages, tumor-associated neutrophils, and natural killer cells) (109). ADPGK-AS1, Mir100hg, and OIP5-AS1 are lncRNAs that are highly expressed in lung cancer samples and are closely associated with disease stage and poor prognosis, suggesting their involvement in metabolic remodeling, immune regulation, and tumor progression (98–100). Within the TME, M2 macrophages—associated with glycolysis—promote tumor growth, whereas M1 macrophages—primarily dependent on OXPHOS—mediate inflammation control (110). ADPGK-AS1 is highly expressed in M2 macrophages and regulates their metabolism and function by interacting with mitochondrial ribosomes, thereby contributing to macrophage phenotypic transformation (98). Simultaneously, the lncRNA Mir100hg, which is found in exosomes released by lung cancer stem cells, alleviates the inhibitory effects of miR-15a-5p and miR-31-5p on glycolysis in tumor cells, thereby enhancing their metastatic potential (99). In addition, OIP5-AS1, present in exosomes in the hypoxic TME of lung adenocarcinoma, affects tumor cell glycolysis via the miR-200c-3p axis, thereby promoting cell proliferation, growth, and metastasis (100). Notably, lncRNAs are also present in exosomes secreted by CAFs. These lncRNAs can downregulate miR-142-5p expression in lung cancer cells and upregulate Programmed cell death ligand 1(PD-L1) on the cell surface, thereby inhibiting the cytotoxic activity of peripheral blood mononuclear cells and facilitating tumor progression (101). Cellular interactions within the TME are not limited to tumor cell-centered regulatory networks. Recent findings reveal an lncRNA-mediated communication axis between CAFs and macrophages: CAF-derived exosomal LINC01833 promotes M2 macrophage polarization in the TME by inhibiting miR-335-5p and modulating VAPA activity (111). Hypoxia-induced HIF-1α also plays a crucial role in the lung cancer TME. Downregulation of miR-214 and upregulation of miR-31-5p lead to increased HIF-1α expression, which subsequently enhances the production of vascular endothelial growth factor. This, in turn, promotes glycolytic metabolic reprogramming (i.e., the Warburg effect) in both cancer cells and TME components, facilitating tumor angiogenesis and proliferation (112).

### 4.2 Lipid metabolism

Lipids perform numerous essential biological functions, including membrane construction, signal transduction, hormone and vitamin synthesis, and energy storage and mobilization during energy deficits (113). In malignant tumor cells, lipid metabolism is reprogrammed primarily to favor endogenous lipid synthesis pathways that utilize glucose and glutamine as substrates to generate citrate through the TCA cycle (114). This process is regulated by central metabolic proteins, such as SREBPs, ACC, FASN, and FABP (114). Aberrations in this pathway may disrupt membrane integrity and permeability, activate apoptosis-related enzymes, and lead to ferroptosis and coproptosis.

**Figure 3:**
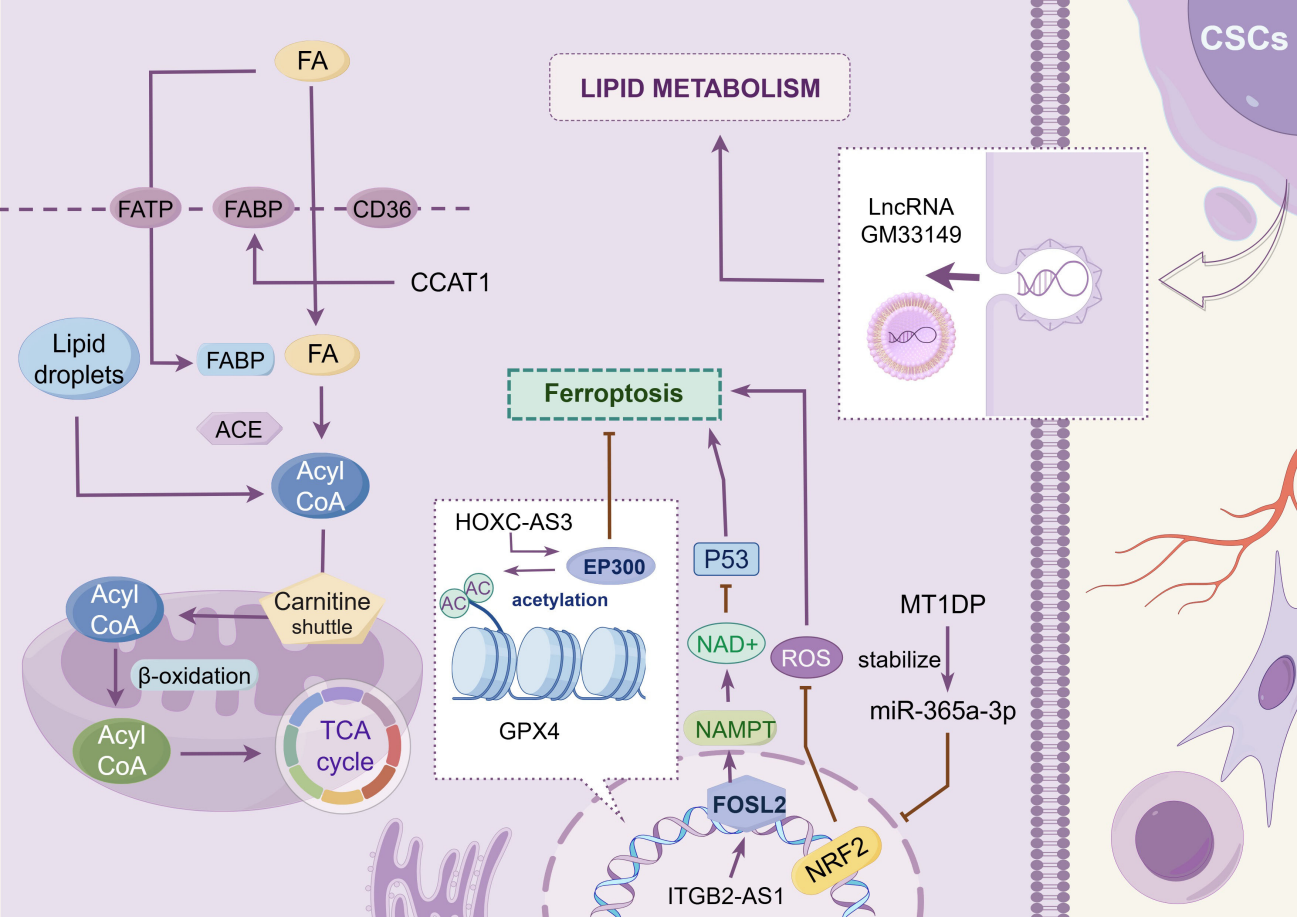
Lipid metabolism. This chart shows the regulatory mechanism of different LncRNAs in the reprogramming of lipid metabolism in tumor cells. These lncRNAs promote the progression of lung cancer by modulating lipid metabolism-related proteins such as FABP5, regulating the expression levels of ferroptosis-related transcription factors such as FOSL2 and NRF2, or facilitating intercellular interactions among tumor cells. The image is created with Figdraw.

Various lncRNAs participate in the regulation of lipid metabolism in lung cancer cells (as shown in **Figure 3** and **Table 2**). Gm33149, localized in the cytoplasm of lung cancer stem cells, is transported to the metastatic microenvironment via microvesicles. As a ceRNA, it binds to miR-5623-3p and miR-217-5p, thereby increasing lipid metabolism in tumor cells with low metastatic potential (115). CCAT1, highly expressed in NSCLC, interacts with FABP5 to enhance fatty acid metabolism (116). It also upregulates BMI-1 expression by targeting miR-218, thereby promoting tumor progression or inducing resistance to gefitinib (120). Ferroptosis contributes to tumor regulation in NSCLC and is orchestrated through key components, including the XC–GSH–GPX4 system and lipid peroxide (PLOOH) synthesis (121). The histone methyltransferase SETD1A and EP300 are co-enriched in the HOXC-AS3 promoter region, promoting the expression of HOXC-AS3 and improving its stability after binding to EP300, thereby inhibiting ferroptosis (117). Nuclear factor erythroid 2-related factor 2 (NRF2) is a transcription factor that protects cells from oxidative stress and promotes malignant transformation (122). MT1DP binds to and stabilizes miR-365a-3p, amplifying its transcriptional inhibitory effect on NRF2, aggravating oxidative damage, and promoting ferroptosis (118). Ye et al. (123) explored the correlation between 13 lncRNAs related to gefitinib metabolism and prognosis, TME, and drug sensitivity in patients with NSCLC. GO and KEGG pathway enrichment analyses suggests that the functions of these lncRNAs are strongly correlated with fat digestion and absorption, as well as α-linolenic acid metabolism, although the specific mechanisms remain to be elucidated.

**Table 2:** Lipid metabolism.

LncRNA	Targets	Downstream component	Deregulation	Functions	References
Gm33149 (ROLLCSC)	miR5623-3p/miR-217-5p	unknown	up	enhance lipid metabolism	(115)
CCAT1	FABP5	CD36/VEGF/PDK1	up	enhance lipid metabolism	(116)
HOXC-AS3	EP300	GPX4	up	inhibit ferropotosis	(117)
MT1DP	miR-365a-3p	NRF2	up	enhance ferropotosis	(118)
ITGB2-AS1	FOSL2	NAMPT/p53	up	inhibit ferropotosis	(119)

### 4.3 Amino acid metabolism

#### 4.3.1 Glutamine metabolism

**Figure 4:**
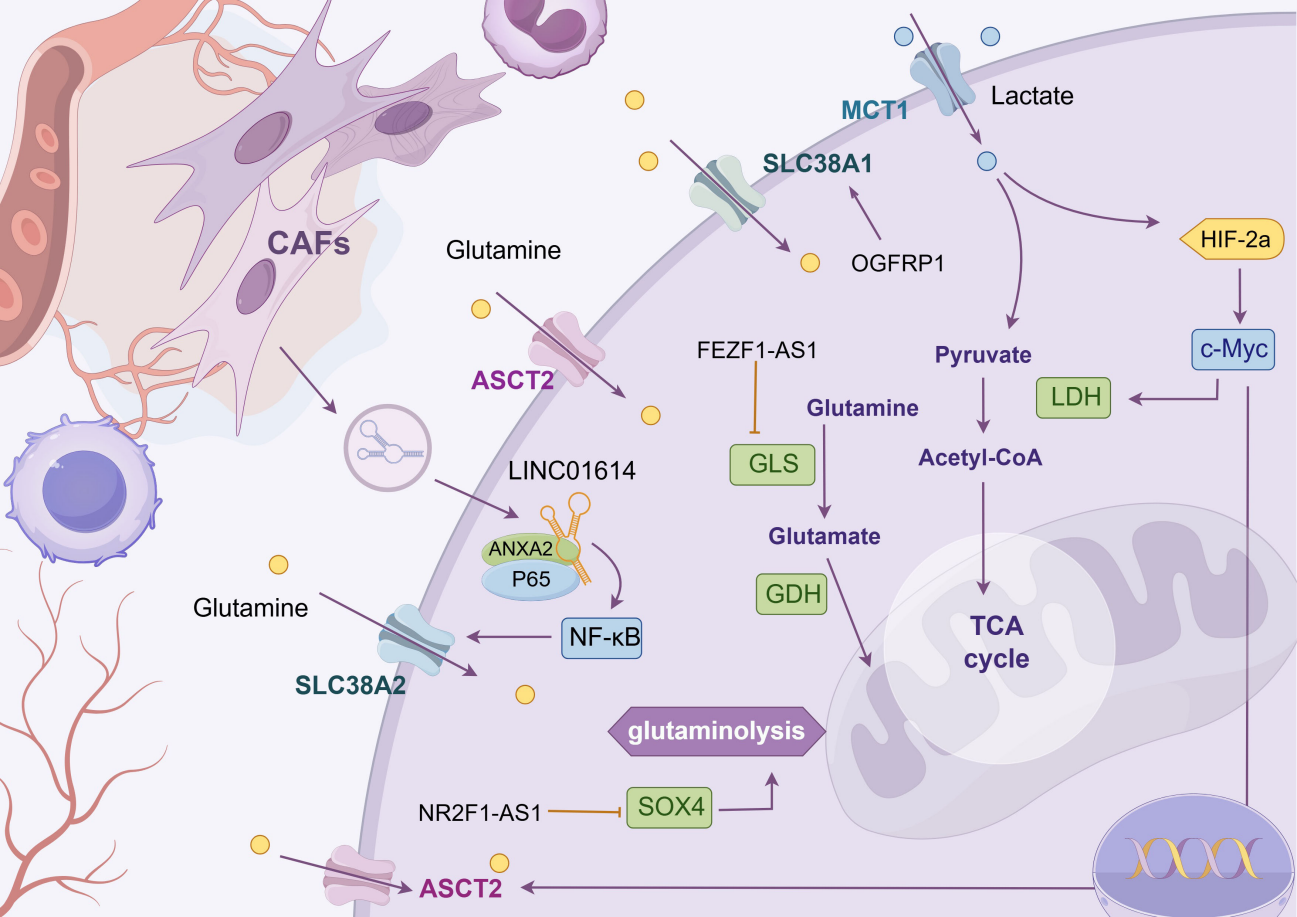
Glutamine metabolism. The chart shows the regulatory mechanism of different LncRNAs in glutamine metabolic reprogramming of tumor cells. LncRNAs can mediate mesenchymal-tumor cell interaction in TME, up-regulate glutamine uptake by cells, or affect the enrichment level of metabolic enzyme GLS to meet the high addiction tendency of lung cancer cells to glutamine. The following is a supplement to the specific mechanisms in the figures: OGFRP1: Indirectly promotes SLC38A1 expression by inhibiting miR-299-3p, alleviating intracellular lipid peroxidation and ferroptosis; FEZF1-AS1: Functions as a “ molecular sponge” to sequester miR-32-5p, enrich GLS, and promote cisplatin resistance in cells; LINC01614: Released from cancer - associated fibroblast exosomes, it interacts with ANXA2 and P65 to activate NF - κ B signaling and initiate the transcription of the glutamine transporters SLC38A2 and SLC7A5; NR2F1-AS1: Highly expressed in non-small cell lung cancer, its silencing reduces HK2 and GLS expression, suppressing glycolysis and glutamine metabolism. Mechanistically, it binds with miR-363-3p to enrich SOX4, promoting glycolysis and glutamine metabolism, and accelerating tumor malignancy. The image is created with Figdraw.

Glutamine, a non-essential amino acid in humans, is synthesized from glucose. However, tumor tissues exhibit a high propensity for glutamine uptake. This phenomenon underscores the significance of glutamine in mammals and its pivotal role in tumor cell proliferation (as shown in **Figure 4** and **Table 3**) (133). In the metabolic reprogramming of lung cancer, the roles of different metabolic axes vary owing to their contributions to tumor growth, drug resistance, and microenvironmental adaptation. Based on existing research, glutamine metabolism may represent the most critical metabolic axis for lung cancer progression, second only to glucose metabolism. Glutamine serves as both a nitrogen and carbon source, supporting intracellular biosynthesis and enhancing the uptake of essential amino acids (134). After entering the cell, glutamine is hydrolyzed by glutaminase (GLS) into glutamic acid, which is then converted into α-ketoglutarate and ammonia by mitochondrial glutamate dehydrogenase (GLUD1) (124); α-ketoglutarate enters the TCA cycle to generate ATP and maintain redox stability, whereas ammonia helps regulate intracellular pH (124).

**Table 3:** Amino acid metabolism.

Classification	LncRNA	Targets	Downstream component	Deregulation	Functions	References
Glutamine metabolism	LINC01614	ANXA2/P65	NF-κB/SLC38A2/SLC7A5	up	increase glutamine intake	(124)
	FEZF1-AS1	miR-32-5p	GLS	up	Promote cisplatin resistance	(125)
	NR2F1-AS1	miR-363-3p	SOX4	up	strengthen glycolysis and glutamine metabolism	(126)
	OGFRP1	miR-299- 3p	SLC38A1	up	inhibit ferropotosis	(126)
Cystine/ Glutamate metabolism	ROR1-AS1	IGF2BP1	SLC7A11	up	inhibit ferropotosis	(127)
	ITGB2-AS1	FOSL2	NAMPT/ p53	up	inhibit ferropotosis	(119)
	T-UCR Uc.339	miR-339	SLC7A11	up	inhibit ferropotosis	(128)
Glycine/ Serine metabolism	MEG8	miR-15a/b-5p	PSAT1	up	promote invasion, proliferation and migration	(129)
	UCA1	miR-495	NRF2	up	promote cisplatin chemoresistance	(130)
	Gm15290	SHMT2	unknown	up	promote proliferation and migration	(131)
Tryptophan metabolism	ZNF8-ERVK3-1	unknown	unknown	up	promote invasion, proliferation and migration	(132)
	MEG3	miR-543	IDO	down	affect immunity and autophagy	(71)

In recent years, numerous studies have demonstrated that lncRNAs regulate glutamine metabolic reprogramming through various mechanisms that promote tumor cell growth and invasion. For example, LINC01614, released from exosomes of CAFs, acts on lung adenocarcinoma cells to activate the NF-κB signaling pathway through interaction with annexin A2 and P65. This activation initiates the transcription of glutamine transporters SLC38A2 and SLC7A5, increasing glutamine uptake by tumor cells (124). In addition, the oncogenic transcription factor c-Myc promotes glutamine catabolism by activating glutamine metabolism genes *SLC1A5* and *GLS1* (135). PCGEM1, a prostate-induced lncRNA, directly interacts with and acts as a coactivator of c-Myc (135). Consequently, we hypothesize that PCGEM1 indirectly regulates glutamine metabolism. FEZF1-AS1, which is upregulated in various tumors, acts as a “molecular sponge” to sequester miR-32-5p, thereby enriching GLS and promoting cisplatin resistance, although the specific mechanism remains unclear (125). In addition, NR2F1-AS1 is highly expressed in NSCLC, and its silencing reduces the expression of HK2 and GLS, thereby inhibiting glycolysis and glutamine metabolic activity (126). Mechanistically, NR2F1-AS1 binds to miR-363-3p to enrich SOX4, promoting glycolysis and glutamine metabolism and accelerating malignant tumor progression (126). Liu et al. demonstrated that the lncRNA OGFRP1 is upregulated in lung cancer cells and indirectly promotes the expression of the glutamine transporter SLC38A1 by inhibiting miR-299-3p, thereby reducing intracellular lipid peroxidation and ultimately suppressing ferroptosis (136). In general, lncRNAs influence glutamine metabolism in tumor cells by regulating the expression of key metabolic enzymes and transporters, thereby supplying the materials and energy required for tumor growth and invasion. Therefore, an in-depth investigation of lncRNA-mediated glutamine metabolic reprogramming may aid in identifying novel tumor biomarkers and therapeutic targets.

Thean abundances of glucose and glutamine in the TME are also crucial for the development and activation of effector T cells. As early as 2018, it was demonstrated that glutamine inhibitors enhance the cytotoxic effect of CD8^+^ T cells (137). Saran et al. (138) further confirmed that the glutamine inhibitor CB-839 inhibits clonal expansion of CD8^+^ T cells in lung adenocarcinoma tissues. They found that activated CD8^+^ T cells depend on glutamine to maintain their antitumor activity and that glutamine inhibitors are currently at the forefront of drug development (139). Their combination with other therapeutic strategies (including radiotherapy and immunotherapy (140)) can amplify antitumor efficacy. CB-839 has advanced to phase I/II clinical trials (141). The application of glutamine inhibitors is not limited to these uses. At present, some researchers (142) have initiated phase I clinical trials in patients with advanced NSCLC, combining the mTOR inhibitor sapanisertib with the glutamine enzyme inhibitor CB-839 to assess drug safety, tolerability, and the recommended dose. However, the current article only reports preliminary findings, such as the scientific rationale, dose-escalation protocols, and cohort stratification criteria. Efficacy results remain pending and require further publication following long-term follow-up.

#### 4.3.2 Cystine and glutamic acid metabolism

The balance of cysteine in cells directly affects the development of ferroptosis (as shown in **Figure 4** and **Table 3**). For example, Jun Deng et al. revealed through experiments that SPTBN2 can increase the expression of the SLC7A11 subunit in the xc(−) system, thereby inhibiting ferroptosis in NSCLC cells and enabling abnormal cell viability (143). The xc(−) system is composed of two important subunits, SLC7A11 and SLC3A2, which are responsible for cystine absorption and glutamate release (11). Extracellular Glu has also been shown to affect the malignant behavior of tumor cells. For instance, Glu promotes the invasion and growth of malignant cells in glioma (144). In the field of cystine and glutamate metabolism in lung cancer, researchers have found that lncRNA ROR1-AS1 is present in the exosomes of CAFs. Its interaction with the N6-methyladenosine reader IGF2BP1 can increase the mRNA stability of SLC7A11, thereby inhibiting ferroptosis (127). Chen et al. were the first to report that ITGB2-AS1, an lncRNA, is abnormally expressed in tumor tissues and cisplatin-resistant tissues of patients with NSCLC. They confirmed that ITGB2-AS1 regulates the expression of NAMPT by interacting with the transcription factor FOSL2, thereby affecting the production of ferroptosis-related proteins, including GPX4 and SLC7A11, with p53 as a downstream target (119). In addition, the lncT-UCRUc.339/miR-339/SLC7A11 axis can serve as a regulatory factor of ferroptosis in lung adenocarcinoma, promoting the proliferation, migration, and invasion of tumor cells (128). Although studies have shown that ITGB2-AS1 can inhibit ferroptosis by regulating SLC7A11, there is no direct evidence that these lncRNA-mediated ferroptosis phenotypes can be reversed *in vivo* by ferroptosis inducers (such as erastin and RSL3). Future research in this direction will help reveal the plasticity of lncRNA–metabolism interactions and provide new avenues for targeted ferroptosis combination therapy.

#### 4.3.3 Glycine and serine metabolism

**Figure 5:**
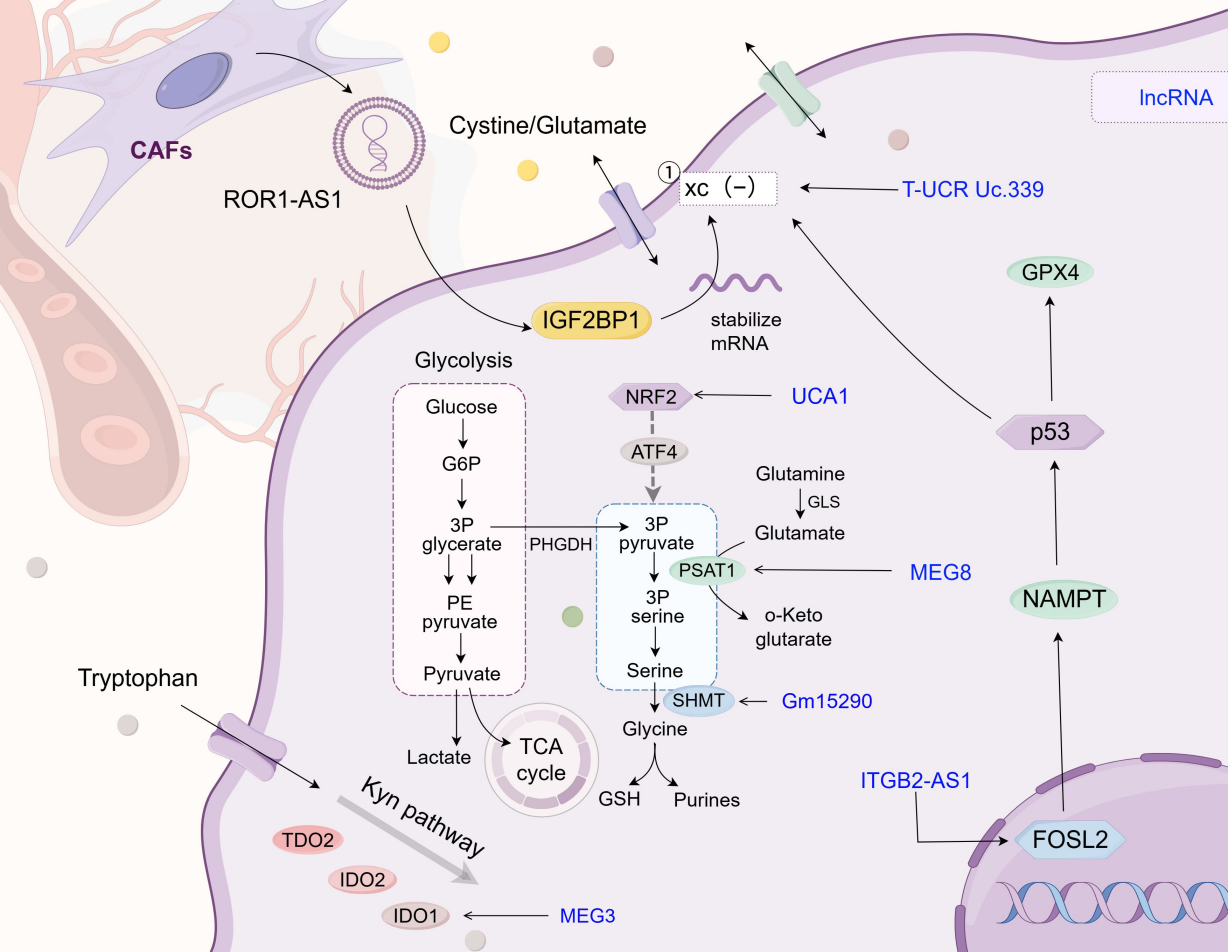
Glycine/serine/tryptophan metabolism. This chart describes the regulatory mechanism of different LncRNAs in the reprogramming of glycine/serine/tryptophan metabolism in tumor cells. Similar to its role in glutamine metabolism, lncRNA also affects the expression level of metabolic enzymes in glycine/serine/tryptophan, maintains the balance of glycine/serine concentration inside and outside the cell, and mediates fibroblast-tumor cell interaction in TME, which also provides novel insights for clinical diagnosis and treatment of lung cancer. The following is a supplement to the specific mechanism shown in the figure: the xc(-) system comprises the SLC3A2 and SLC7A11 subunits; The lnc T-UCR Uc.339/miR-339/SLC7A11 axis serves as a factor influencing ferroptosis in lung adenocarcinoma and promotes the proliferation, migration, and invasion of tumor cells; LncRNA UCA1 targets miR-495 to indirectly regulate NRF2 expression in cisplatin-resistant NSCLC cells; LncRNA MEG8 drives NSCLC progression *in vivo* via the miR-15a/b-5p/PSAT1 axis; In lung cancer cells, lncRNA Gm15290 mediates serine - to - glycine conversion via SHMT2 and is regulated by lncRNA Gm15290 as a downstream target; ITGB2-AS1 is aberrantly expressed in the tumor tissue and cisplatin-resistant tissue of NSCLC patients. It regulates NAMPT expression by interacting with transcription factor FOSL2 and affects the production of ferroptosis - related proteins, including GPX4 and SLC7A11, by targeting p53 downstream; In NSCLC, LncRNA MEG3 is low - expressed. It can bind miR - 543 as a ceRNA and affects autophagy of lung cancer cells by regulating the IDO pathway; LncRNA ROR1-AS1 exists in cancer-associated fibroblast (CAF) exosomes, interacts with the N6-methyladenosine (m6A) reader IGF2BP1 to enhance SLC7A11 mRNA stability, and suppresses ferroptosis. The image is created with Figdraw.

Glycine in cells can be converted from an intermediate product of glucose glycolysis, 3-phosphoglycerate. This pathway is also involved in serine synthesis. Following transamination and dephosphorylation, serine is synthesized by transferring a one-carbon unit to the folate pool (145). Gene set enrichment analysis has revealed that the transcription factor NRF2 may play a key regulatory role in the PHGDH and serine biosynthesis pathways (146). Based on this finding, researchers also discovered that NRF2 can induce the expression of proteins required for serine biosynthesis in an ATF4-dependent manner, such as PHGDH, phosphoserine aminotransferase 1 (PSAT1), PSPH, serine hydroxymethyltransferase (SHMT) 1, and SHMT2 (146). The lncRNA MEG8 promotes NSCLC progression through the miR-15a/b-5p/PSAT1 axis *in vivo*, further corroborating the key role of PSAT1 in NSCLC development (129). SHMT2 mediates the conversion of serine to glycine in lung cancer cells, and it can be regulated by lncRNA Gm15290 as its downstream target (131) (as shown in **Figure 5** and **Table 3**).

In cisplatin-resistant NSCLC cells, UCA1 targets miR-495 to indirectly modulate NRF2 expression, although this process does not involve direct detection of serine/glycine metabolism (130). Shi et al. revealed that UCA1 regulates HO1 expression through NRF2 to enhance chemotherapy resistance in lung adenocarcinoma cells; however, the effect of lncRNA UCA1 on serine/glycine metabolism requires further verification (147).

#### 4.3.4 Tryptophan metabolism

Tryptophan is an essential amino acid for humans, and its metabolites have been shown to be closely associated with the progression of various tumors (as shown in **Figure 5**; **Table 3**) (148). Tryptophan is catalyzed by the IDO1, IDO2, and TDO2 enzyme families in the kynurenine pathway to produce ATP, the redox cofactor NAD^+^, and metabolites, such as nicotinic acid, palmitate, quinolinic acid, and serotonin. IDO1 degrades tryptophan to generate the immunosuppressive metabolite kynurenine, which induces T-cell depletion and promotes the differentiation of regulatory T cells, thereby establishing an immunosuppressive microenvironment (149). Moreover, PD-1 can inhibit regulatory T cell activation by targeting CD28 costimulatory signals (150). These findings suggest that targeting the lncRNA-mediated IDO1 pathway may synergize with PD-1/PD-L1 inhibitors. However, this hypothesis remains to be confirmed by further experimental data.

In lung cancer, gastric cancer, colorectal cancer, breast cancer, and other malignancies, circulating tryptophan levels are generally reduced, potentially because of increased expression and activity of metabolic enzymes, as well as the nutritional status and dyspepsia of patients in advanced stages (148). In addition, Gao et al. used bioinformatic approaches to identify that the lncRNA ZNF8-ERVK3-1 in lung adenocarcinoma cells is closely associated with tryptophan metabolism and confirmed that it can promote the proliferation, migration, and invasion of lung adenocarcinoma cells (132). However, the underlying mechanisms remain to be elucidated. Lu et al. further confirmed that lncRNA MEG3 is expressed at low levels in NSCLC and that MEG3 can act as a ceRNA to bind miR-543 and affect autophagy in lung cancer cells by regulating the IDO pathway (71). Collectively, these findings suggest that targeting lncRNAs involved in the IDO pathway of tryptophan metabolism may offer a novel immunotherapeutic strategy for the treatment of lung cancer.

## 5 Discussion

The lncRNA family constructs a complex regulatory network by adsorbing miRNAs and regulating cellular behavior at the transcriptional, posttranscriptional, and protein levels, providing important guidance for the development of targeted therapeutic drugs. Recent studies have revealed remarkable survival characteristics of cancer cells. Fundamental tumor characteristics, including metabolic reprogramming, are involved in various biological processes, such as drug resistance, cell proliferation, cell death, and oxidative stress homeostasis. These findings highlight the need to consider the influence of multiple factors on malignant behavior during diagnosis and treatment and offer potential explanations and coping strategies for individual differences in clinical efficacy.

Currently, the primary methods of lung cancer treatment include surgery, radiotherapy, chemotherapy, targeted therapy, and immunotherapy. However, each modality has its own strengths and limitations and is often used in combination. With the discovery and validation of novel oncogenic lncRNAs, biomarkers for the early diagnosis of lung cancer have been continuously enriched. Some lncRNAs also serve as potential therapeutic targets for NSCLC. For example, lncLCETRL3/4 drives resistance to tyrosine kinase inhibitors in EGFR-mutant NSCLC by activating the AKT signaling pathway. Combined targeting of lncLCETRL3/4 and tyrosine kinase inhibitors may significantly reverse drug resistance and improve therapeutic efficacy (151). Targeted protein degradation strategies based on lncRNAs are highly effective in reducing the malignant phenotype of cancer cells. Cao et al. (152) developed artificial “alncRNAs” that can target and promote the degradation of carcinogenic transcription factors, such as c-MYC, NF-κB, and EGFR. This approach has demonstrated therapeutic effects, including inhibition of proliferation, migration, and invasion and promotion of apoptosis in bladder cancer cells.

The regulatory mechanisms of lncRNAs provide a foundation for the development of clinically targeted drugs. Efforts are underway to develop lncRNA-targeted therapies or to employ RNA interference techniques, such as siRNA and antisense oligonucleotides (ASOs), to downregulate oncogenic lncRNAs (153, 154). Some lncRNAs have already entered preclinical research or early clinical trials. For instance, preclinical studies have shown that targeted inhibition of lncRNA MALAT1 by ASOs can effectively reduce lung cancer metastasis in mouse models (155). LncRNA PVT1 is upregulated in NSCLC tissues, and its knockout significantly inhibits cancer cell viability and invasion in xenograft mouse models while inducing apoptosis (156). In clinical trials, downregulation of HOTAIR has been shown to reduce the expression of DNA methyltransferases and increase the sensitivity of SCLC cells to cisplatin, adriamycin, and etoposide (156). Additionally, H19-DTA (BC-819), a therapeutic strategy targeting lncRNA H19, offers a promising new option for the treatment of breast, lung, and other malignancies. Phase I/II clinical trials (NCT04691739) for bladder and ovarian cancers are currently underway, with plans to extend this approach to lung cancer treatment in the future (157). These advancements offer substantial insights for the clinical translation of lncRNA-based therapies. From a technical perspective, ASOs and siRNAs targeting oncogenic lncRNAs have demonstrated potent antitumor effects. CRISPR-based gene editing technologies have shown unique advantages in preclinical models, offering high specificity, long-term silencing, and lower off-target risks (158). For example, the CRISPR-Cas9 system has been used to inhibit the expression of ΔNp63 in lung cancer, significantly suppressing the proliferation of lung squamous cell carcinoma cells, inducing apoptosis, and effectively blocking tumor growth in xenograft mouse models (159). CRISPR interference has demonstrated a more durable silencing effect than that of traditional ASO/siRNA approaches, although its delivery system still requires optimization. Therefore, the combined application of CRISPR and ASO/siRNA may become a key strategy to overcome therapeutic bottlenecks in future tumor treatments. Moreover, with the advent of big data, new personalized therapies based on patients’ lncRNA expression profiles have begun to emerge, aiming to predict prognosis and identify therapeutic targets (26).

Although lncRNAs have shown great promise in the regulation of lung cancer metabolism, several challenges continue to hinder their clinical translation: (1) Current research on lncRNA- and miRNA-mediated metabolic reprogramming in lung cancer is primarily based on *in vitro* cell lines (such as A549 and H1299), which cannot fully replicate the complexity of the TME *in vivo (* 160). (2) Only a limited number of lncRNA-targeted therapies have advanced to phase I clinical trials, suggesting a substantial gap between laboratory findings and clinical application (161). (3) The development of both animals and plants relies on lncRNAs to regulate epigenetics through dynamic modular structures and target specificity. However, challenges remain because of the complexity of protein interaction networks and accuracy of target recognition. These issues must be addressed using multi-omics techniques, such as iCLIP 422, RAP-MS 423, ChIRP-MS 388, and iDRiP (162). (4) Despite the utility of current methods, comprehensive analyses of lncRNA subcellular localization, structural characteristics, and dynamic interaction networks remain technically challenging. For example, while RNA-FISH technology enables the localization of lncRNAs, its resolution is limited (163). Similarly, although CLIP-seq can capture lncRNA–protein interactions, it is susceptible to interference from RNA secondary structures and low-abundance transcripts (164).

The clinical translation of lncRNA-targeted therapies is particularly constrained because lncRNAs, as large, negatively charged molecules, are inherently difficult to transport across cell membranes. Existing delivery systems, such as lipid nanoparticles, struggle to achieve efficient and targeted intracellular delivery, and it is difficult to accurately direct them to specific tissues or organs, which limits the therapeutic efficacy of lncRNAs *in vivo*. Zhang et al. (165) used lentiviral vectors to achieve stable overexpression of lncRNAs. However, lncRNAs overexpressed in cell lines often contain additional sequences that alter their secondary structures, impair RNA-protein interactions, prevent them from exerting their intended functions, and may also be recognized by the patient’s immune system as exogenous molecules, activating immune receptors, such as TLRs, inducing inflammatory responses or immunotoxicity, and thereby affecting the safety and tolerability of treatment (166). Additionally, lncRNA therapy may lead to off-target effects because of sequence similarity or excessive administration, resulting in unintended actions on non-target cells or tissues, which can compromise therapeutic efficacy and increase potential risks (152, 166). It is evident that lncRNA-targeted therapy faces severe challenges in clinical translation, fundamentally stemming from the contradiction between the molecular characteristics of lncRNAs and complex physiological environment *in vivo*, and further research is needed to bridge these gaps. In addition to the lncRNAs discussed in this article, research on other noncoding RNAs, such as circRNAs, is also progressing and is expected to further expand the frontiers of disease diagnosis and treatment (167). Looking ahead, with the continued advancement of ncRNA research, more accurate and efficient strategies for cancer diagnosis and therapy are anticipated.

In summary, studies of the lncRNA family have elucidated the core mechanisms underlying cancer cell survival and development, providing novel insights for personalized and precise treatment. In the future, with the advancement of technology and a deeper understanding of the mechanisms of ncRNAs, further breakthroughs in the field of cancer therapy are expected, bringing renewed hope to patients.
